# Association between dietary flavonoid intakes and C-reactive protein levels: a cross-sectional study in Taiwan

**DOI:** 10.1017/jns.2021.8

**Published:** 2021-03-04

**Authors:** Cheng-Tzu Hsieh, Jui Wang, Kuo-Liong Chien

**Affiliations:** 1 Institute of Epidemiology and Preventive Medicine, College of Public Health, National Taiwan University, Taipei 10055, Taiwan; 2 Department of Internal Medicine, National Taiwan University Hospital, Taipei, Taiwan

**Keywords:** C-reactive protein, Cross-sectional study, Flavonoids, Flavonoid-rich foods, BMI, body mass index, CI, confidence interval, CRP, C-reactive protein, DBP, diastolic blood pressure, FDB-EXP, USDA's Expanded Flavonoid Database for the Assessment of Dietary Intakes, HDL, high-density lipoprotein, LDL, low-density lipoprotein, NAHSIT 2005-8, Nutrition and Health Survey in Taiwan 2005–2008, OR, odds ratio, SBP, systolic blood pressure

## Abstract

Although the intake of specific flavonoid-rich foods may reduce C-reactive protein (CRP) levels, the association between dietary flavonoid intakes and CRP is inconsistent. We aim to describe dietary flavonoid intakes in a Taiwanese nationally representative sample and to investigate the association between flavonoid intakes and CRP. We conducted a cross-sectional study based on 2592 adults from the Nutrition and Health Survey in Taiwan 2005–8. Flavonoid intakes were estimated by linking the 24-h dietary recall with the U.S. Department of Agriculture flavonoid database and divided into quartiles. Adjusted estimates of the flavonoid intakes for the continuous and binary (elevated CRP: >0⋅3 mg/dl) variables were performed by using general linear and logistic regression. We found that tea, orange, tofu and sweet potato leaves/water spinach constituted the major food items of the total flavonoid intake. The total flavonoid intake was lower among women and elderly. Compared with the lowest total flavonoid intake quartile, participants in higher quartiles were associated with a lower CRP status (adjusted odds ratio (OR): 0⋅61, 95 % confidence interval (CI): 0⋅44–0⋅86 for the highest quartiles). The trends were similar for flavonol and flavan-3-ol intakes. Compared with non-consumers, tea consumers were likely to have a lower CRP status (adjusted OR: 0⋅74, 95 % CI: 0⋅57–0⋅97). In brief, a higher total flavonoid intake and tea consumption were inversely associated with CRP levels, indicating that a high-flavonoid diet may contribute to anti-inflammatory effects. A Taiwanese flavonoid content table is necessary for conducting further studies related to flavonoids in Taiwan.

The inflammatory process has been recognised to play a key role in the pathogenesis of cardiovascular diseases (CVD)^(1)^. C-reactive protein (CRP), an acute-phase reactant of inflammation synthesised by the liver, was identified as a marker of inflammation. A high level of CRP was thought to associate with future CVDs^(2,3)^.

Previous studies have shown that higher intakes of vegetables, fruits^(4,5)^ and tea^(6,7)^ were associated with lower CRP levels. The mechanisms responsible for the benefits and the specific constituents of vegetables, fruits and tea that may explain these associations are unclear; however, flavonoids, the largest subgroup of polyphenols distributed in plants, may play an important role^(8)^ in these associations.

Flavonoids have been proved to be effective antioxidants for their abilities to clean reactive oxygen species, inhibit lipid peroxidation and protect free radical-induced damage to DNA^(8,9)^. More than 5000 flavonoid compounds have been described, and they can be divided into six major subclasses, namely, flavonols, flavones, flavanones, flavan-3-ols, anthocyanidins and isoflavones^(8)^.

Although several studies have estimated the major sources of flavonoid intakes^(10–17)^ and have investigated the association between dietary total flavonoid intake and CPR levels^(18–20)^, most of the studies were conducted in the Western countries and their results are still inconsistent. In addition, the dietary habits among people differed in Asian and Western countries^(21–23)^. As diverse flavonoid compounds are found in different foods and the antioxidative properties of each flavonoid compound are different, we were wondering whether higher total flavonoid intakes based on Asia dietary habits were also inversely associated with CRP levels. Therefore, the objectives of the present study are to describe the dietary flavonoid intakes of a Taiwanese nationally representative sample and to examine the associations of dietary flavonoid intakes and flavonoid-rich food consumption with CRP levels.

## Methods

### Nutrition and Health Survey in Taiwan 2005–8

In this cross-sectional study, we used data collected from the Nutrition and Health Survey in Taiwan 2005–8 (NAHSIT 2005–8). The NAHSIT 2005–8 was the third period of the NAHSIT, and the target population were Taiwanese nationals aged 0–6 years (0 year was defined as aged from birth to less than 1 year) and 19 years and above. The survey adopted a multi-stage sampling scheme. The 358 townships/city districts and the three ethnic minority groups in Taiwan were divided into eight sampling strata (five strata for geographical location and three for ethnic minority groups). The probability proportional to size sampling methods was used to select six townships or city districts for each of the eight strata. Among each of the forty-eight selected townships, two sampling blocks were selected to generate sample lists. Information pertaining to socio-demographics, lifestyle, 24-h dietary recall and health-related questionnaires was collected within households by conducting face-to-face interviews. Health examinations, including physical examinations, which included the collection of blood samples and urine samples, were conducted in the temporarily established clinics. Seasonal effects were taken into account using the Latin square design to control seasonal variation that may affect dietary consumption and nutritional status. Detailed information on the NAHSIT 2005–8 survey has been published elsewhere^(24)^.

In the present study, we analysed data collected from the individuals of the NAHSIT 2005–8 aged 19 years and older. There were some stipulations: all participants in this study should have an appropriate 24-h dietary recall (500–5000 kcal/d). The participants who were included in the analyses related to CRP should further possess data on CRP concentration.

### Food consumption data

We estimated the dietary data from the NAHSIT 2005–8 24-h dietary recall. Details of food consumption in the past 24 h were collected by conducting face-to-face interviews. To ensure the quantity of foods consumed, food-piece models (for dishes containing chopped, sliced or shredded foods), multiple hollow hemisphere models (for round-shaped foods) for Taiwanese and other standard cooking measures were used^(25)^. The 24-h dietary recall data included the weights of all the consumed food sources from each participant. Moreover, the Food Consumption Database of Taiwan was used to group all the food sources into 290 food items. In this study, we used these 290 food items to describe the dietary intakes.

### U.S. Department of Agriculture flavonoid databases

The ‘U.S. Department of Agriculture (USDA)'s Expanded Flavonoid Database for the Assessment of Dietary Intakes (FDB-EXP)’ was used to establish the flavonoid content table. The FDB-EXP was published by the USDA, Agricultural Research Service in September 2014. The FDB-EXP contained data for twenty-nine flavonoid compounds in six classes for 2926 foods. The following are the twenty-nine flavonoid compounds: flavonols (isorhamnetin, kaempferol, myricetin and quercetin), flavones (apigenin and luteolin), flavanones (eriodictyol, hesperetin and naringenin), flavan-3-ols (catechin, epicatechin, epicatechin-3-gallate, epigallocatechin, epigallocatechin-3-gallate, gallocatechin, theaflavin, theaflavin-3-gallate, theaflavin-3′-gallate, theaflavin-3,3′-digallate and thearubigins), anthocyanidins (cyanidin, delphinidin, malvidin, pelargonidin, peonidin and petunidin) and isoflavones (daidzein, genistein and glycitein)^(26)^.

### Estimation of flavonoid intakes

We linked the NAHSIT 2005–8 24-h dietary recall data with the FDB-EXP to estimate the flavonoid intakes (Supplementary Fig. S1 of Supplementary material). First, we divided the twenty-nine flavonoid compounds in the FDB-EXP into six subclasses, making six individual flavonoid groups (flavonols, flavones, flavanones, flavan-3-ols, anthocyanidins and isoflavones). The content of individual flavonoids for each food source in the FDB-EXP was the summation of all the flavonoid compounds belonging to the same subclass. Secondly, we divided all the food sources in the FDB-EXP into 290 food items using the Food Consumption Database of Taiwan. The content of the individual flavonoid for each of the 290 food items was the median from the food sources that belong to the same food item. The total flavonoids for each food item were the summation of six individual flavonoids. Taking ‘apple’ as an example, we found that there were nine different kinds of apples in the FDB-EXP. We grouped these nine apples into an ‘apple item’ based on the Food Consumption Database of Taiwan. Also, the content of flavonols for the ‘apple item’ was the median of the flavonols from the nine different kinds of apples. The content of the other five flavonoid subclasses was defined by the same method. The total flavonoids for the ‘apple item’ were the summation of six individual flavonoids.

Next, dietary individual flavonoid intakes from selected food items were determined by multiplying the content of the individual flavonoids by the dietary consumption of the selected food items. Finally, the total intake of individual flavonoids was the sum of individual flavonoid intakes from all food items reported in the 24-h dietary recall. Also, the total flavonoid intake was determined by the summation of the total intake of individual flavonoids.

### CRP measurement

CRP concentrations were obtained from 8 h fasting blood samples in the NAHSIT 2005–8. CRP concentrations were measured using a particle-enhanced immunoturbidimetric test with an automatic analyser (Hitachi 747, Japan). The limit of detection was 0⋅07 mg/dl; the intra- and inter-assay CV were less than 10 %^(27)^. The value of CRP >0⋅3 mg/dl was considered as elevated CRP concentration^(28)^.

### Confounding factors

We collected information about the smoking status, drinking status, physical activities and education levels of the participants from questionnaires. Smoking and drinking status were divided into yes (current and ex-smoker/ex-drinker) or no (never). Physical activities were divided into yes (more than 30 min/week) or no (less than 30 min/week). Education levels were divided into three groups: elementary or below, high school and university or above. We obtained data on age, gender, body mass index (BMI), systolic blood pressure (SBP), diastolic blood pressure (DBP), glucose, triacylglycerol, total cholesterol, low-density lipoprotein (LDL) cholesterol and high-density lipoprotein (HDL) cholesterol from physical examinations. BMI was calculated as weight in kilograms divided by the square of height in metres. Information on total energy intakes and vitamin C and vitamin E intakes were provided by the NAHSIT 2005–8 database, which relied on the Nutrient Composition Database for Foods in Taiwan.

### Statistical analyses

Total and individual flavonoid intakes were considered as continuous variables and were divided into quartiles. As the first and second quartiles of flavanones were both 0, we divided flavanone intake into less than the third quartile group (≤Q3) and more than the third quartile group (>Q3). Similarly, the first quartile of anthocyanidins was 0; therefore, we divided anthocyanidin intake into low (less than the second quartile), middle (the second to the third quartile) and high (more than the third quartile) groups. CRP was considered as continuous and binary (>0⋅3 mg/dl as elevated CRP) variables.

To estimate the major food items of dietary total flavonoid intake, we calculated the contribution of each food item to the total flavonoid intake from all participants by taking the proportion of dietary total flavonoids provided by the specific food item over the total flavonoids from all food items.

The median and the interquartile ranges were used to describe the total and individual flavonoid intakes. Mann–Whitney *U* test and Kruskal–Wallis test were used to compare the differences according to gender and age groups (19–44, 45–64 and ≥65 years old).

The Student's *t* test, analysis of variance (ANOVA) and the *χ*^2^ tests were used to compare means and proportions among the quartiles of flavonoid intakes. The quartiles of flavonoid intakes were entered as dummy variables into regression models. Multiple linear regression was used to estimate the association between flavonoid intakes and CRP concentrations. Multiple logistic regression was applied to calculate the odds ratios (OR) and 95 % confidence intervals (CIs) of elevated CRP. To investigate the associations, we adjusted for confounding factors as follows. In model 1, we adjusted for gender (women or men), age (a continuous variable), BMI (a continuous variable), smoking status (yes or no), drinking status (yes or no), education levels (elementary or below/high school/university or above), physical activities (yes or no) and total energy intake (a continuous variable). Model 2 was constructed from model 1 and included SBP, DBP, glucose, triacylglycerol, total cholesterol, LDL cholesterol and HDL cholesterol. Model 3 was constructed from model 2 and included vitamin C (a continuous variable) and vitamin E (a continuous variable) intakes, and the two antioxidants found in vegetables and fruits.

We further investigated the associations of flavonoid-rich food consumptions with elevated CRP using logistic regression adjusted for potential confounding factors. Tea, oranges, tofu and sweet potato leaves/water spinach, the top four major food items of total flavonoids, were divided into two categories of non-consumers and consumers. Moreover, legumes, fruits and vegetables, the food groups that have been known as the major flavonoid sources, were divided into non-consumers, low consumers (less than the third quartile) and high consumers (more than the third quartile).

Moreover, in order to test whether total flavonoid intake was associated with elevated CRP independent from tea, fruit or vegetable consumption, the mutually adjusted models were used to combine the total flavonoid intake and tea, fruit, or vegetable consumption into the same models.

All statistical tests were two-tailed, and values of *P* < 0⋅05 were taken to indicate statistical significance. All analyses were performed using SAS 9.4 software (SAS Institute, Inc., Cary, NC, USA).

### Ethical standards disclosure

This study was conducted according to the guidelines laid down in the Declaration of Helsinki, and all procedures involving research study participants were approved by the ethics committees of the National Taiwan University Hospital (201702037RINB). Written informed consent was obtained from all subjects.

## Results

### Study participants

A total of 6214 individuals were recruited in the NAHSIT 2005–8. In the present study, we excluded the individuals without 24-h dietary recall and those who had unappropriate total energy intake for 24-h dietary recall (<500 kcal/d or >5000 kcal/d). We also excluded individuals who were below 19 years old. A total of 4508 participants were included in the analyses for major food items of dietary total flavonoid intake. Among the 4508 participants, 1916 did not have available CRP measurements; thus, the remaining 2592 eligible participants were included in the other analyses (Supplementary Fig. S2 of Supplementary material).

### Major food items of dietary total flavonoid intake

The major food items according to gender and age groups (19–44, 45–64 and ≥65 years old) are shown in Table 1. The top four food items were similar among different gender and age groups. The major food item was tea, which provided about 90 % of the total flavonoid intake. Although the order of items was slightly different according to gender and age, oranges, tofu and sweet potato leaves/water spinach were the second to fourth food items of the total flavonoid intake.

**Table 1.** Major food items of dietary total flavonoid intake, specified by gender and age groups

	Gender				Age groups					
	Males (*n* 2244)		Females (*n* 2264)		19–44 years old (*n* 1513)		45–64 years old (*n* 1492)		≥65 years old (*n* 1503)
	Food items	%	Food items	%	Food items	%	Food items	%	
1	Tea	93⋅95	Tea	86⋅25	Tea	92⋅57	Tea	92⋅33	Tea	88⋅56
2	Tofu	0⋅59	Oranges	1⋅43	Tofu	0⋅81	Oranges	0⋅76	Sweet potato leaves/water spinach	1⋅25
3	Oranges	0⋅45	Sweet potato leaves/water spinach	1⋅13	Oranges	0⋅47	Tofu	0⋅61	Oranges	1⋅20
4	Sweet potato leaves/water spinach	0⋅45	Tofu	1⋅01	Sweet potato leaves/water spinach	0⋅44	Sweet potato leaves/water spinach	0⋅54	Tofu	0⋅76
5	Bamboo shoot	0⋅29	Other fruits and vegetables^a^	0⋅82	Soya milk	0⋅41	Soya milk	0⋅39	Other fruits and vegetables^a^	0⋅73

a Other fruits and vegetables: eggplant, daylily-bud, okra, Roselle, etc.

### Individual and total flavonoid intakes

Compared with men, women had lower total flavonoid (the median was 99⋅8 mg/d for men and 55⋅2 mg/d for women) intakes. Compared with younger adults, participants older than 65 years had lower total flavonoid (the median was 79⋅0 mg/d for 19–44 years old, 86⋅7 mg/d for 45–64 years old and 52⋅1 mg/d for ≥65 years old) intakes. There were major differences in both flavonol and flavan-3-ol intakes. The six individual flavonoid and total flavonoid intakes according to gender and age groups (*n* = 2592) are shown in Table 2.

**Table 2.** Individual and total flavonoid intakes according to gender and age groups

Median (interquartile range)	Gender			Age groups			
	Males (*n* 1266)	Females (*n* 1326)	*P*	19–44 years old (*n* 771)	45–64 years old (*n* 886)	≥65 years old (*n* 935)	*P*
Flavonols (mg/d)	27⋅5 (68⋅9)	17⋅8 (30⋅2)	<0⋅001	21⋅3 (47⋅2)	23⋅3 (49⋅7)	18⋅5 (38⋅7)	<0⋅001
Flavones (mg/d)	0⋅7 (1⋅7)	0⋅8 (1⋅7)	0⋅69	0⋅5 (1⋅4)	0⋅8 (1⋅7)	0⋅8 (2⋅0)	<0⋅001
Flavanones (mg/d)	0⋅0 (0⋅4)	0⋅0 (1⋅1)	0⋅34	0⋅0 (0⋅2)	0⋅0 (2⋅5)	0⋅0 (0⋅5)	0⋅010
Flavan-3-ols (mg/d)	19⋅7 (1139⋅2)	7⋅6 (29⋅6)	<0⋅001	16⋅1 (748⋅1)	14⋅8 (647⋅7)	6⋅1 (36⋅6)	<0⋅001
Anthocyanidins (mg/d)	0⋅2 (2⋅9)	0⋅4 (3⋅3)	0⋅25	0⋅4 (3⋅0)	0⋅5 (4⋅1)	0⋅1 (2⋅3)	<0⋅001
Isoflavones (mg/d)	0⋅3 (14⋅4)	0⋅2 (13⋅6)	0⋅08	0⋅3 (18⋅7)	0⋅3 (16⋅3)	0⋅2 (9⋅1)	<0⋅001
Total flavonoids (mg/d)	99⋅8 (1228⋅3)	55⋅2 (133⋅7)	<0⋅001	79⋅0 (823⋅6)	86⋅7 (690⋅0)	52⋅1 (163⋅4)	<0⋅001

### Basic characteristics of participants

The basic characteristics of participants according to the total flavonoid intake are shown in Table 3. Compared with the lowest quartile, participants in higher quartiles were more likely to be younger; more likely to have a lower waist circumference, SBP, DBP, glucose, and triacylglycerol and more likely to have a higher HDL cholesterol, total energy intake, vitamin C intake and vitamin E intake. Regarding the categorical variables, the participants in the higher quartiles were more likely to be men, were more likely to smoke and consume alcohol, and also more likely to have higher education levels.

**Table 3.** Basic characteristics according to quartiles of total flavonoid intake

	Q1		Q2		Q3		Q4		
Range (mg/d)	≤28⋅7		28⋅7–68⋅7		68⋅7–557⋅8		>557⋅8		
*n*	650		642		652		648		
	Mean values	sd	Mean values	sd	Mean values	sd	Mean values	sd	*P*
Age (years)	57⋅6	18⋅7	54⋅0	17⋅8	53⋅7	16⋅4	51⋅6	16⋅9	<0⋅001
Waist (cm)	84⋅1	11⋅2	82⋅4	10⋅9	82⋅3	10⋅7	84⋅3	10⋅6	<0⋅001
BMI (kg/m^2^)	24⋅7	4⋅2	24⋅3	4⋅1	24⋅4	4⋅0	24⋅7	3⋅7	0⋅17
SBP (mmHg)	123⋅2	20⋅3	118⋅8	19⋅1	118⋅2	19⋅2	118⋅9	17⋅8	<0⋅001
DBP (mmHg)	71⋅4	12⋅1	71⋅1	11⋅4	71⋅1	11⋅0	73⋅0	11⋅2	0⋅005
Glucose (mg/dl)	115⋅3	44⋅6	109⋅9	33⋅9	109⋅7	35⋅5	110⋅6	34⋅3	0⋅019
Triacylglycerol (mg/dl)	134⋅2	107⋅6	133⋅3	105⋅0	122⋅6	78⋅8	137⋅6	105⋅7	0⋅041
Total cholesterol (mg/dl)	192⋅7	41⋅2	194⋅3	38⋅8	192⋅5	35⋅6	192⋅4	38⋅2	0⋅78
HDL cholesterol (mg/dl)	53⋅0	15⋅6	54⋅5	14⋅6	55⋅3	15⋅9	51⋅1	14⋅8	<0⋅001
LDL cholesterol (mg/dl)	120⋅8	37⋅1	122⋅8	36⋅8	122⋅1	33⋅6	122⋅5	34⋅9	0⋅78
Total energy (kcal/d)	1453⋅5	651⋅0	1710⋅4	748⋅4	2054⋅2	832⋅6	2032⋅3	886⋅4	<0⋅001
Vitamin C (mg/d)	101⋅4	97⋅9	148⋅7	128⋅0	232⋅1	185⋅6	207⋅4	171⋅8	<0⋅001
Vitamin E (mg/d)	5⋅9	4⋅9	7⋅7	5⋅4	10⋅2	7⋅0	9⋅0	6⋅7	<0⋅001
	*n*	%	*n*	%	*n*	%	*n*	%	*P*
Females	377	58⋅0	377	58⋅7	367	56⋅3	205	31⋅6	<0⋅001
Smoker	174	28⋅3	177	29⋅8	161	25⋅7	307	49⋅8	<0⋅001
Drinker	243	39⋅6	260	43⋅7	253	40⋅4	355	57⋅4	<0⋅001
Education									<0⋅001
Elementary or below	380	58⋅5	285	44⋅5	203	31⋅1	181	27⋅9	
High school	201	30⋅9	281	43⋅9	347	53⋅2	357	55⋅1	
University or above	69	10⋅6	74	11⋅6	102	15⋅6	110	16⋅9	

BMI, body mass index; SBP, systolic blood pressure; DBP, diastolic blood pressure; HDL, high-density lipoprotein; LDL, low-density lipoprotein; Q, quartile; sd, standard deviation.

### Association between total/individual flavonoid intakes and CRP

**Fig. 1.**
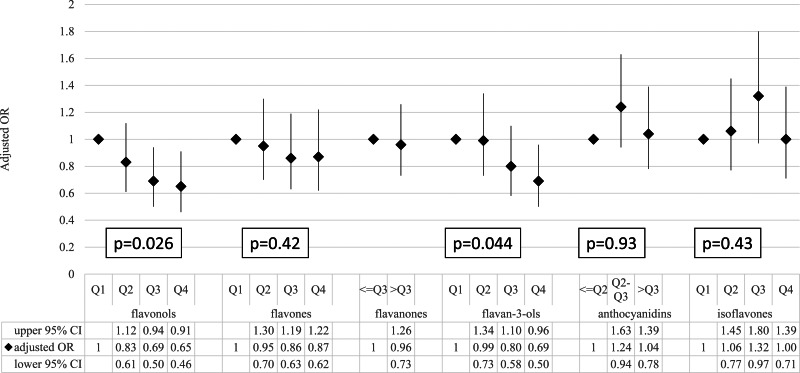
The association between individual flavonoid intakes and the risk of elevated C-reactive protein. The ORs and 95 % CIs were estimated by using the logistic regression model adjusted for age, gender, BMI, smoking status, drinking status, education levels, physical activities, total energy, SBP, DBP, glucose, triacylglycerol, total cholesterol, LDL cholesterol, HDL cholesterol, and vitamin C and vitamin E intakes. CI, confidence interval; OR, odds ratio;*P*, *P* for trend test; Q, quartile.

The association of the total flavonoid intake with the status of elevated CRP is shown in Table 4 (the results of linear regression are shown in Supplementary Table S1 of Supplementary material). Compared with the lowest total flavonoid intake quartile (reference group), participants in higher quartiles were more likely to have a lower CRP status (adjusted OR: 0⋅80 (95 % CI: 0⋅59–1⋅09), 0⋅68 (95 % CI: 0⋅48–0⋅95) and 0⋅61 (95 % CI: 0⋅44–0⋅86) from the second quartile to the highest quartile, respectively; *P* for trend 0⋅039). The trends were similar for flavonol and flavan-3-ol intakes (*P* for trend 0⋅026 for flavonols and 0⋅044 for flavan-3-ols, using logistic regression based on a full model). However, no association was found for flavone, flavanone, anthocyanidin and isoflavone intakes (Fig. 1).

**Table 4.** The association of total flavonoid intake with the status of elevated C-reactive protein

	Q1	Q2		Q3		Q4		
Range (mg/d)	≤28⋅7	28⋅7–68⋅7		68⋅7–557⋅8		>557⋅8		
Median (mg/d)	15⋅5	45⋅8		119⋅2		1610⋅5		
*n*	650	642		652		648		
Elevated CRP	161	114		98		101		
	Odds ratios	Odds ratios	95 % confidence intervals	Odds ratios	95 % confidence intervals	Odds ratios	95 % confidence intervals	*P*
Model 1	1	0⋅71	0⋅53–0⋅96	0⋅58	0⋅42–0⋅80	0⋅54	0⋅39–0⋅75	0⋅015
Model 2	1	0⋅78	0⋅57–1⋅05	0⋅62	0⋅45–0⋅86	0⋅58	0⋅41–0⋅80	0⋅019
Model 3	1	0⋅80	0⋅59–1⋅09	0⋅68	0⋅48–0⋅95	0⋅61	0⋅44–0⋅86	0⋅039

CRP, C-reactive protein; Q, quartile.

Model 1: Logistic regression was used to adjust for age, gender, BMI, smoking status, drinking status, education levels, physical activities and total energy.

Model 2: Model 2 was constructed from model 1, and it included additional adjustments for SBP, DBP, glucose, triacylglycerol, total cholesterol, LDL cholesterol and HDL cholesterol.

Model 3: Model 3 was constructed from model 2, and it included additional adjustments for vitamin C and vitamin E intakes.

### Association between flavonoid-rich food intakes and CRP

We included the top four major food items of total flavonoid intakes (tea, oranges, tofu and sweet potato leaves/water spinach) as well as the three food groups that have been known as the major flavonoid sources (legumes, fruits and vegetables) in the analyses of the associations between flavonoid-rich food intakes and CRP levels.

Compared with non-consumers, tea consumers tend to have a lower risk of elevated CRP (adjusted OR: 0⋅74, 95 % CI: 0⋅57–0⋅97). However, trends were not found for orange (adjusted OR: 1⋅36, 95 % CI: 0⋅82–2⋅22), tofu (adjusted OR: 1⋅23, 95 % CI: 0⋅89–1⋅70) and sweet potato leaves/water spinach (adjusted OR: 0⋅89, 95 % CI: 0⋅67–1⋅19) consumers (Table 5).

**Table 5.** The association of flavonoid-rich foods consumption with C-reactive protein concentrations and risk of elevated C-reactive protein

	Non-consumers (elevated CRP)	Consumers (elevated CRP)	Adjusted odds ratios (consumers *v.* non-consumers)	95 % confidence intervals
Tea	1849 (361)	743 (113)	0⋅74	0⋅57–0⋅97
Oranges	2455 (446)	137 (28)	1⋅36	0⋅83–2⋅22
Tofu	2254 (411)	338 (63)	1⋅23	0⋅89–1⋅70
Sweet potato leaves/ water spinach	2097 (389)	495 (85)	0⋅89	0⋅67–1⋅19

Adjusted for age, gender, BMI, smoking status, drinking status, education levels, physical activities, total energy, SBP, DBP, glucose, triacylglycerol, total cholesterol, LDL cholesterol, HDL cholesterol, and vitamin C and vitamin E intakes.

As for the results of the flavonoid-rich food groups (Supplementary Table S2 of Supplementary material), higher fruit and vegetable consumption was associated with a lower risk of elevated CRP. The adjusted OR was 0⋅66 (95 % CI: 0⋅47–0⋅94; *P* for trend 0⋅021) for a high-consumed fruit group and was 0⋅36 (95 % CI: 0⋅18–0⋅71; *P* for trend 0⋅045) for a high-consumed vegetable group. However, no association was found for legume consumption (adjusted OR 0⋅87, 95 % CI: 0⋅65–1⋅16).

### Mutually adjusted models

In the mutually adjusted models (Supplementary Table S3 of Supplementary material), we combined the total flavonoid intake and tea, fruit or vegetable consumption into the same logistic regression model (a full model). The results remained significant in the total flavonoid/fruit adjusted model (*P* for trend 0⋅022 and 0⋅012 for total flavonoid intake and fruit consumption) and in the total flavonoid/vegetable adjusted model (*P* for trend 0⋅032 and 0⋅036 for total flavonoid intake and vegetable consumption). These results may imply that higher flavonoid intakes were associated with lower CRP levels, and this association may not be due to other components in fruits or vegetables. However, the results in the total flavonoid/tea adjusted model became non-significant (*P* for trend 0⋅77 and 0⋅95 for total flavonoid intake and tea consumption). Although further studies are necessary to clarify whether there is any direct association between tea consumption and flavonoid intake, it was possible that the benefits of tea consumption may stem from the flavonoids in tea.

## Discussion

In this study, we linked the NAHSIT 2005–8 24-h dietary recall data with the FDB-EXP to estimate the flavonoid intakes. Tea was the major source of total flavonoid intake, following by oranges, tofu and sweet potato leaves/water spinach. We also found that the total flavonoids, flavonols and flavan-3-ols from Taiwanese dietary habits were inversely associated with CRP levels.

In our study, the four major flavonoid sources were tea, oranges, tofu and sweet potato leaves/water spinach. In the USA, the major food sources of flavonoid intakes were investigated in 1999–2002 and 2007–10^(14,17)^ using a 24-h dietary recall. The major dietary sources of total flavonoid intake were similar in the two periods among U.S. adults: they consisted of tea, citrus fruit juices (primarily including orange juices), wine and citrus fruits (primarily including oranges). A study that used data from the Nurses’ Health Study cohort also found similar results, and the major food items were tea, apples and citrus fruits (including juices)^(29)^. One study that used data from the TwinsUK registry found that tea constituted 81 % of the total flavonoid intakes^(20)^. In addition, a study that used the data from the Australia National Nutrition Survey 1995 found the items tea, oranges, other citrus fruits, grapes and wine in descending order in the total flavonoid intake^(10)^. Based on these studies, it can be found that tea is the major item in regard to the total flavonoid intake: approximately 90 % in this study, 53–82⋅8 % in the USA, 81 % in the UK and 76 % in Australia.

Several previous studies have investigated the associations between total and individual flavonoid intakes and CRP levels in the USA and the UK, but the results are still inconsistent. Two studies conducted in the USA using the National Health and Nutrition Examination Survey 1999–2002 presented inconsistent conclusions regarding the association between total flavonoid intake and CRP levels. The first study conducted in 2008 concluded that total flavonoid intake was inversely associated with CRP levels, and the CRP geometric means for the non-consumers and the third quartile were 2⋅69 and 1⋅84 mg/l, respectively, *P* for trend < 0⋅05^(18)^. The second study in 2011 did not find any significant results between total flavonoid intake and elevated CRP (adjusted OR of the fifth quintile 0⋅89; 95 % CI: 0⋅70–1⋅13)^(19)^. However, the point estimate of less than 1 still implies the potential preventive effects of high total flavonoid intake on elevated CRP. These inconsistent results may be due to the adoption of different statistical methods or different grouping strategies. In addition, two studies using the 131-item food frequency questionnaire to assess flavonoid intakes did not find the association between total flavonoid intake and CRP levels^(20,29)^. Both studies included twenty flavonoid compounds to estimate the total flavonoid intake. In the present study, we used the 24-h recall to estimate the twenty-nine flavonoid compound intakes. We found an inverse association between total flavonoid intake and CRP levels. The 24-h recall enables us to have details of food consumption, and the broad range of flavonoid compounds enables us to estimate the total flavonoid intake more accurately. We believed that high total flavonoid intake may have preventive effects on high CRP levels. Further studies that can estimate dietary flavonoid intake with a more complete flavonoid content table are necessary to confirm the effects of flavonoid intake on CRP levels.

Our study found that tea consumption was associated with lower CRP levels, which was consistent with previous studies^(6,7)^. Flavonoid compounds were considered the major active compounds present in teas^(30)^. Several cell culture and animal studies have shown that flavonoid compounds in teas can inhibit the formation of reactive oxygen and regulate the gene expression and transcription for inflammation^(31,32)^. Therefore, teas can be considered as a healthy drink to reduce CRP levels.

Oranges were important sources of total flavonoid intake, but their consumption was not associated with CRP levels in our study. An intervention study found that the CRP concentrations tended to be lower but not significant after drinking 500 ml/d of high-pressurised orange juice for 14 d^(33)^. One possible reason is that the two major flavonoid compounds in citrus fruits, hesperetin and naringenin, are considered weaker antioxidants^(34)^.

Tofu and sweet potato leaves/water spinach were the common foods in Asia, and both were important sources of flavonoids in our study. However, we found that no study had investigated the associations of the consumption of tofu and sweet potato leaves/water spinach with CRP levels. One study among premenopausal women showed that the 2-year soya intervention, including tofu, soya milk, roasted soya nuts, soya bars and soya protein powder, was not associated with CRP levels^(35)^. As for sweet potato leaves/water spinach, only one study has shown that sweet potato leaves have a high antioxidant activity *in vitro*^(36)^. Further studies are warranted to investigate the benefits of these important flavonoid sources in Asia.

Flavonoids may decrease oxidative stress in the phospholipid bilayer by trapping the chain-initiating radicals at the interface of the membranes^(8)^. Flavonoids are also shown to inhibit the synthesis and gene expression of cytokines. Nuclear factor (NF)-κB is an oxidant sensitive upstream regulator of proinflammatory mediator synthesis. Oxidative stress is an inducer of NF-κB. When the activated NF-κB translocates into the nucleus and binds to NF-κB-responsive genes, an inflammatory cascade is triggered and CRP is subsequently produced^(37)^. Flavonoids are thought to inhibit the formation of CRP by blocking the activation of NF-κB and to inhibit the binding with genes^(9,38,39)^.

In this study, we found that a higher dietary flavonoid intake may contribute to the anti-inflammatory effect and, therefore, may decrease the risk of CVD. We suggested that higher dietary vegetables, fruits and tea, which were rich in flavonoids, should be encouraged. In addition, in order to provide appropriate suggestions for a flavonoid-rich diet to the people in Asia, further studies about the benefits of flavonoid-rich foods in Asia, such as tofu and sweet potato leaves/water spinach, are warranted.

Our study had several limitations. First, as a cross-sectional design, the flavonoid intake and CRP level were simultaneously assessed, and no data could provide evidence for a temporal relationship between the two. However, CRP is not a common biomarker for the public, flavonoids are not well-known nutrients and nutritional supplements for flavonoids are rare^(40)^. People are less likely to adjust their dietary habits based on their CRP levels. Secondly, we estimated the dietary flavonoid intakes based on one 24-h dietary recall, which is known to have a high within-person variability. However, the 24-h dietary recall is a good tool to assess dietary intakes in a population^(41)^. Thirdly, self-reported dietary intakes were used to measure food intakes in this study, and the actual intakes of some healthy food items such as vegetables may be lower than reported^(42)^. Therefore, the flavonoid intakes in this study may be overestimated. If such overestimation had indeed occurred while assessing the vegetable intakes of people with poor dietary habits, and if we had assumed that people with poor dietary habits had poor health status, the results of our study may only lead to a null result. Fourthly, we focused only on flavonoid intake but not on the bioavailability and metabolism of flavonoids in human subjects. Therefore, we may not be aware of the antioxidant effects of flavonoids on people after the consumption of flavonoid-rich foods and whether higher flavonoid intakes are associated with better antioxidant effects. Fifthly, the results of our study may not directly reveal that the association between flavonoid-rich food consumption and CRP levels is directly related to flavonoids. However, the results still provide hints for further studies about the possible roles played by flavonoids in vegetables, fruits and tea. Finally, we used the FDB-EXP to create the flavonoid content table, which may not cover all the food sources in the NAHSIT 2005–8, especially for soya and soya products. Therefore, the soya-related flavonoid, called isoflavones, may be underestimated in our study. A Taiwanese database similar to the FDB-EXP is necessary for conducting further studies in Taiwan.

In conclusion, tea was the major source of dietary total flavonoid intakes among participants of the NAHSIT 2005–8. We demonstrated the inverse association of flavonol, flavan-3-ol and total flavonoid intakes with CRP levels, implying that a flavonoid-rich diet may significantly produce anti-inflammatory effects. A Taiwanese flavonoid content table is necessary for conducting further studies related to flavonoids in Taiwan.

## Supplementary material

For supplementary material accompanying this paper visit https://doi.org/10.1017/jns.2021.8. click here to view supplementary material
