# Supplementary material for: Association between dietary flavonoid intakes and C-reactive protein levels: a cross-sectional study in Taiwan
Source: J Nutr Sci. 2021 Mar 4;10:e15. doi: 10.1017/jns.2021.8 (PMC8057367; doi:10.1017/jns.2021.8)
Supplement: Supplementary file 1 [file S2048679021000082sup001.docx]

**(Online Supporting Material) Supplemental Figure 1: Flavonoids intake measurement from USDA’s Expanded Flavonoid Database for the Assessment of Dietary (FDB-EXP) and the Nutrition and Health Survey 2005-2008 (NAHSIT 2005-2008) in Taiwan.**

USDA’s Expanded Flavonoid Database for the Assessment of Dietary (FDB-EXP)

Dividing the 29 flavonoid compounds into six subclasses

(6 individual flavonoids)

The content of individual flavonoid for each food source (mg/100g food) = the summation of all the flavonoid compounds belong to the same subclass

Grouping all the food sources into 290 food items using Food Consumption Database of Taiwan

The content of individual flavonoid for each food item (mg/100g food item) = the medians of the individual flavonoid from the food sources that belong to the same food item

Total flavonoids for each food item= the summation of individual flavonoids

Nutrition and Health Survey in Taiwan 2005-2008 24-hour dietary recall

Listing all the foods sources in the 24-hour diet recall

Calculating the weight of consumption from each food item for each participant (g/food item/day)

Dietary individual flavonoid intake from each food item= multiplied the content of the individual flavonoid (mg/100g food item) by the dietary consumption of each food item (g/day)

Total intake of individual flavonoids=the summation of individual flavonoid intakes from all food items reported in the 24- hour dietary recall

Total flavonoids intake= the summation of total intake of individual flavonoids

Estimating the contribution of each food item to the daily total intake of flavonoids for all participants

Linking with NAHSIT questionnaires and physical examinations data for further analysis

Grouping all the food sources into 290 food items using Food Consumption Database of Taiwan

Provided by NAHSIT

**(Online Supporting Material) Supplemental Figure 2: Flow diagram.**

**6104** participants with 24-h dietary recall data

**6214** participants in NAHSIT

**5896** participants with appropriated total energy intake for 24-h dietary recall

<19 years old

(n=1388)

**4508** adults

No 24-hour dietary recall data (n=110)

24-hour dietary recall <500kcal/day (n=136)

24-h dietary recall >5000kcal/day (n=72)

**2592** eligible participants

No CRP measurement (n=1916)

**4508** participants: estimated the contribution of food items to the daily total intake of flavonoids

**(Online Supporting Material) Supplemental Table 1:** The association of total flavonoids intake with C-reactive protein concentrations

|  | Q1 | Q2 |  | Q3 |  | Q4 |  |  |
| --- | --- | --- | --- | --- | --- | --- | --- | --- |
| Range, mg/day | ≤28.7 | 28.7-68.7 |  | 68.7-557.8 |  | >557.8 |  |  |
| n | 650 | 642 |  | 652 |  | 648 |  |  |
|  |  | Estimated parameter | SEM | Estimated parameter | SEM | Estimated parameter | SEM | test for trend |
| Model 1 | reference | -0.108 | 0.038 | -0.161 | 0.039 | -0.156 | 0.040 | 0.036 |
| Model 2 | reference | -0.081 | 0.037 | -0.137 | 0.039 | -0.141 | 0.039 | 0.026 |
| Model 3 | reference | -0.078 | 0.038 | -0.131 | 0.040 | -0.136 | 0.040 | 0.040 |

Abbreviation: CI, confidence interval; CRP, C-reactive protein; OR, odds ratio; Q, quartile; SEM, standard error of the mean;

Model 1: General linear regression were used to adjust for age, gender, body mass index, smoking status, drinking status, education levels, physical activities, and total energy.

Model 2: Model 2 was constructed from model 1, and it included additional adjustments for systolic blood pressure, diastolic blood pressure, glucose, triglyceride, total cholesterol, low-density lipoprotein cholesterol, and high-density lipoprotein cholesterol.

Model 3: Model 3 was constructed from model 2, and it included additional adjustments for vitamin C and vitamin E intakes.

**(Online Supporting Material) Supplemental Table 2: The association between legumes, fruits, and vegetables consumption and the risk of elevated CRP.**

|  | Non-consumers | Low  (0-Q3) |  | High  (>Q3) |  |  |
| --- | --- | --- | --- | --- | --- | --- |
| Legumes, g/day | 0 | 0-83.1 |  | >83.1 |  |  |
| Fruits, g/day | 0 | 0-246.4 |  | >246.4 |  |  |
| Vegetables, g/day | 0 | 0-475.6 |  | >475.6 |  |  |
|  | OR | OR | 95% CI | OR | 95% CI | test for trend |
| Legumes | 1 | 1.21 | 0.93-1.58 | 0.87 | 0.65-1.16 | 0.18 |
| Fruits | 1 | 0.77 | 0.59-1.00 | 0.66 | 0.47-0.94 | 0.021 |
| Vegetables | 1 | 0.44 | 0.23-0.84 | 0.36 | 0.18-0.71 | 0.045 |

Abbreviation: CI, confidence interval; OR, odds ratio; Q, quartile;

The ORs and 95% CIs were estimated by logistic regression model adjusted for age, gender, body mass index, smoking status, drinking status, education levels, physical activities, total energy, systolic blood pressure, diastolic blood pressure, glucose, triglyceride, total cholesterol, low-density lipoprotein cholesterol, high-density lipoprotein cholesterol, and vitamin C and vitamin E intakes.

**(Online Supporting Material) Supplemental Table 3: Combination of total flavonoids intake and tea, fruits or vegetables consumption adjusted for confounding factors in multivariable-adjusted models.**

|  | Total flavonoids intake | | |  | Flavonoids-rich food consumption | | |
| --- | --- | --- | --- | --- | --- | --- | --- |
|  | Q4  OR | 95% CI | test for trend |  | Q3  OR | 95% CI | test for trend |
| Total flavonoids/Tea | 0.70 | 0.11-4.60 | 0.77 |  | 0.88 | 0.14-5.62 | 0.95 |
| Total flavonoids/Fruits | 0.63 | 0.45-0.89 | 0.022 |  | 0.69 | 0.48-0.99 | 0.012 |
| Total flavonoids/Vegetables | 0.63 | 0.45-0.88 | 0.032 |  | 0.39 | 0.19-0.77 | 0.036 |

Abbreviation: CI, confidence interval; OR, odds ratio; Q, quartile;

The ORs and 95% CIs were estimated by logistic regression model adjusted for age, gender, body mass index, smoking status, drinking status, education levels, physical activities, total energy, systolic blood pressure, diastolic blood pressure, glucose, triglyceride, total cholesterol, low-density lipoprotein cholesterol, high-density lipoprotein cholesterol, and vitamin C and vitamin E intakes.

OR and 95% CI values were for the highest compared with the lowest group (quartile for total flavonoids intake; non-consumers for food consumption)
